# The ‘pay-to-publish’ model should be abolished

**DOI:** 10.1098/rsnr.2016.0039

**Published:** 2016-08-24

**Authors:** Raghavendra Gadagkar

**Affiliations:** Centre for Ecological Sciences and Centre for Contemporary Studies, Indian Institute of Science, Bangalore, 560012, India President, Indian National Science Academy, Bahadur Shah Zafar Marg, Delhi, 110002, India

Scholarly journals have become increasingly expensive and unaffordable, whether for individuals or for libraries. This has been exacerbated by the enormous increase in the numbers of published articles and therefore in the per-capita consumption necessary for scholarly activity. The arrival of digital publishing and the Internet have magnified these numbers almost beyond imagination.

Funders of scholarly activity, be they government or private, have begun to complain that they have to pay twice—first to produce the research and then to read it, while profits go largely to commercial publishers. It therefore appears that the traditional ‘publish-for-free and pay-to-read’ model will no longer work. It has appeared for some time that there is a simple alternative—flip the model and make it ‘pay-to-publish and read-for free’. Buoyed by the politically attractive label ‘open access’, this model has spread very rapidly and taken on many forms.

I argue that the ‘pay-to-publish and read-for-free’ model (hereafter ‘pay-to-publish’ model) has far more serious problems. Including publication costs within research grants is being widely advocated and implemented but it seems to be not so widely recognized that funders still pay twice—first to produce the research and then to publish it, and profits still go largely to commercial publishers. Since funders pay twice in the old model and continue to pay twice in the new model, one might be tempted to think that at least the new model is not any worse than the old one. But it is much worse, for at least two reasons.

First, the pay-to-publish model makes the playing field even more uneven for scholars; those from less well-endowed institutions and poor countries will suffer even more because the quantum of grants required to do research and publish it is now greater than it was before. As I have argued in more detail elsewhere, poor countries and poor scholars will be doomed to remain knowledge consumers (since they can read-for-free) rather than become knowledge producers (since they have to pay-to-publish)—generating and perpetuating a form of knowledge hegemony incompatible with self-respect and equal participation.^1^

Second, and perhaps even more serious, the ‘pay-to-publish’ model is inherently unstable. In the language of evolutionary biology, it is ‘susceptible to cheating’. Nothing prevents unscrupulous publishers from publishing trash as long as the authors pay for it. This is not a fanciful prophecy—it is a growing reality having already attained frightening proportions, enough to warrant the recognition of a new genre of ‘predatory journals’. A recent study revealed that 420 000 articles were published in what have been termed ‘fake’ journals, at an average price of $178 per article, in the year 2014 alone.^2^ The profits of such journals are estimated to be of the order of $75 million a year. These numbers may be mind-boggling but this is only the tip of the iceberg—runaway selection can easily swamp the genuine articles into oblivion.

Is there a solution? Yes, and I suggest a twofold solution. First, the required fraction of research funds (whatever be the size of the total pie, and whoever pays it) should be set aside for subsidizing the publication of a new model of ‘publish-for-free and read-for-free’ journals by scholarly societies, academies and other ‘not-for-profit’ organizations. Only the remaining fraction of the pie should be made available for doing research. It is important to emphasize that the money set aside for publication should not be given to individual researchers to buy their way into publication: it should be given only to the ‘not-for-profit’ organizations that will not charge authors. There are many ways of organizing the disbursement of funds meant for publishing and I do not wish to narrow the basket of possibilities, except to argue strongly for promoting many diverse and decentralized ‘not-for-profit’ publishing ventures.

Second, and equally importantly, the ‘pay-to-publish’ model should be dismantled altogether. We should gradually create social and moral stigma, and eventually legal strictures, against paid publications; having paid for publishing scholarly papers should automatically devalue their prestige and eventually disqualify them from consideration.

These two steps I believe could rescue the scientific journal from its imminent end.

## Notes

1 Raghavendra Gadagkar, ‘Open-access more harm than good in developing world’, *Nature* **453**, 450 (2008); Raghavendra Gadagkar, ‘Open access does more harm than good when based on a “pay to publish” business model’, *Physiology News* **75**, 46 (2009).

2 http://news.sciencemag.org/education/2015/09/predatory-publishers-earned-75-million-last-year-study-finds (accessed 30 June 2016).

